# Evaluating clinical effectiveness of 13-valent pneumococcal conjugate vaccination against pneumonia among middle-aged and older adults in Catalonia: results from the EPIVAC cohort study

**DOI:** 10.1186/s12879-018-3096-7

**Published:** 2018-04-27

**Authors:** Angel Vila-Corcoles, Olga Ochoa-Gondar, Cinta de Diego, Eva Satue, María Aragón, Angel Vila-Rovira, Frederic Gomez-Bertomeu, Ramon Magarolas, Enric Figuerola-Massana, Xavier Raga, Mar O. Perez, Frederic Ballester

**Affiliations:** 1 Primary Care Service Camp de Tarragona, Institut Catala de la Salut, Tarragona, Spain; 2 Unitat de Suport a la Recerca of Tarragona, Institut Universitari d’Investigació en Atenció Primària Jordi Gol (IDIAP Jordi Gol), Tarragona, Spain; 3 Information System for the Improvement of Research in Primary Care (SIDIAP), Primary Care Research Institute Jordi Gol, Universitat Autonoma de Barcelona, Barcelona, Spain; 4 Primary Care Research Institute Jordi Gol, Barcelona, Spain; 5 Department of Laboratory and Microbiology, Hospital Joan XXIII, Tarragona, Spain; 6 Department of Pneumology, Hospital Joan XXIII, Tarragona, Spain; 7 Department of Otolaryngology, Hospital Joan XXIII, Tarragona, Spain; 8 Department of Laboratory and Microbiology, Hospital Santa Tecla, Tarragona, Spain; 9 Department of Laboratory and Microbiology, Hospital de Tortosa Verge de la Cinta, Tortosa, Tarragona, Spain; 10 Department of Laboratory and Microbiology, Hospital Sant Joan de Reus, Tarragona, Spain

**Keywords:** Adults, Effectiveness, Pneumococcal conjugate vaccine, Pneumonia, Streptococcus pneumoniae

## Abstract

**Background:**

Benefits using the 13-valent pneumococcal conjugate vaccine (PCV13) in adults are controversial. This study investigated clinical effectiveness of PCV13 vaccination in preventing hospitalisation from pneumonia among middle-aged and older adults.

**Methods:**

Population-based cohort study involving 2,025,730 individuals ≥50 years in Catalonia, Spain, who were prospectively followed from 01/01/2015 to 31/12/2015. Primary outcomes were hospitalisation for pneumococcal or all-cause pneumonia and death from any cause. Cox regression models were used to evaluate the association between PCV13 vaccination and the risk of each outcome, adjusting for age, sex and major comorbidities/underlying risk conditions.

**Results:**

Cohort members were observed for a total of 1,990,701 person-years, of which 6912 person-years were PCV13 vaccinated. Overall, crude incidence rates (per 100,000 person-years) were 82.8 (95% confidence interval [CI]: 77.7–88.1) for pneumococcal pneumonia, 637.9 (95% CI: 599.0–678.7) for all-cause pneumonia and 2367.2 (95% CI: 2222.8–2518.7) for all-cause death. After multivariable adjustments we found that the PCV13 vaccination did not alter significantly the risk of pneumococcal pneumonia (multivariable-adjusted hazard ratio [mHR]: 1.17; 95% CI: 0.75–1.83; *p* = 0.493) and all-cause death (mHR: 1.07; 95% CI: 0.97–1.18; *p* = 0.190), although it remained significantly associated with an increased risk of all-cause pneumonia (mHR: 1.69; 95% CI: 1.48–1.94; *p* < 0.001). In stratified analyses focused on middle-aged or elderly persons and immunocompromised or immunocompetent subjects, PCV13 vaccination did not appear effective either.

**Conclusion:**

Our data does not support clinical benefits of PCV13 vaccination against pneumonia among adults in Catalonia. It must be closely monitored in future studies involving more vaccinated person-time at-observation.

## Background

Pneumococcal infections are a major cause of morbidity and mortality, especially among infants, high-risk adults and elderly people [1]. Among adults, apart from the classical 23-valent pneumococcal polysaccharide vaccine (PPsV23), [2] a 13-valent protein-polysaccharide conjugate vaccine (PCV13) has been available for use in adults since 2012 [3].

At present, the Advisory Committee on Immunization Practices (ACIP) of the Centers for Disease Control and Prevention (CDC, Atlanta, Ga, USA) recommends PCV13 vaccination (sequentially with the PPsV23) for those persons aged 19–64 years who have high-risk conditions (mainly anatomical or functional asplenia and immunocompromising conditions) and all persons 65 years or older (with or without risk conditions) [4, 5]. These recommendations are based on immunogenicity studies (which showed that PCV13 induced an immune response as good as or better than that induced by PPsV23 for common serotypes) [6, 7] and the results of the *CAPITA* study (a randomised-controlled trial [RCT] comparing PCV13 vs placebo among elderly individuals in the Netherlands) [8] which reported a PCV13 efficacy of 46% against vaccine-type invasive pneumococcal infections. However, some experts and clinicians are not in accordance with a positive interpretation of data reported in the *CAPITA* trial and disagree with ACIP’S recommendations [9–11].

In Catalonia, a region in Northeastern Spain with approximately seven million people, the PPsV23 has been recommended for high-risk and older adults since the 2000s, reaching a coverage of approximately 60–70% in these persons [12]. The PCV7/PCV13 have been used for childhood immunisation since they were marketed in 2001 and 2010, respectively, reaching intermediate vaccination coverage (48% in at-risk infants) [13]. Among adults, the PCV13 is recommended and publicly funded for some high-risk individuals (basically immunocompromised patients), [14] being also prescribed by some clinicians for adults with other risk conditions (chronic pulmonary or respiratory disease, heart disease, liver disease, diabetes mellitus, etc) although it is not publicly funded in these patients and, consequently, PCV13 coverage remains low [12].

Considering this, we designed a population-based cohort study, known as *EPIVAC* (Effectiveness of Pneumococcal and Influenza Vaccinations among Adults in Catalonia), with the major aim of evaluating possible clinical benefits of antipneumococcal and influenza vaccinations among the general population over 50 years. In this report we have analysed the clinical effectiveness of PCV13 vaccination in preventing hospitalisation for pneumonia (pneumococcal and all-cause) in the study cohort at one-year follow-up. In addition, considering the debate about current PCV13 recommendations, we have also assessed vaccination effectiveness in stratified analyses according to age subgroups and immunological situation.

## Methods

### Design, setting and study population

This is a closed population-based prospective cohort study involving 2,025,730 middle-aged and older adults in Catalonia, Spain. Cohort members were all persons assigned to the 274 Primary Health Care Centres (PHCCs) managed by the Catalonian Health Institute (Institut Catala de la Salut, ICS) who were aged 50 years or older at December 31, 2014.

**Fig. 1:**
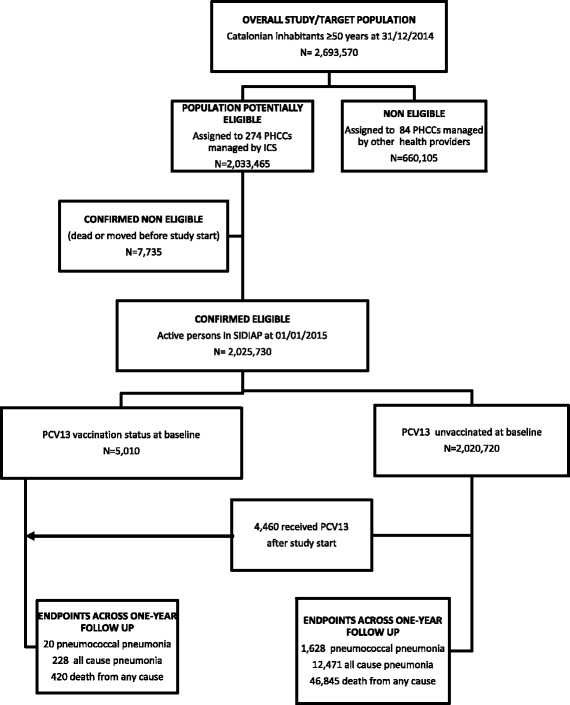
Number of individuals at each stage of the study, vaccination status and endpoints across one-year follow-up

In the study setting, similar to the rest of Spain, all inhabitants are covered by the National Health Service by a compulsory health assurance system; so, all inhabitants are assigned to a PHCC. Around Catalonia there are 358 PHCCs, of which 274 (76.5%) are managed by the ICS and 84 PHCCs are managed by other providers. The analysed cohort (*n* = 2,025,730 persons) represents a 75.2% of the total 2,693,570 Catalonian inhabitants over 50 years-old according to 2014 census data (Fig. 1).

Cohort members were followed since the beginning of the study (01/01/2015) until the occurrence of any event, disenrollment from the PHCC, death, or until the end of one-year follow-up (31/12/2015).

The study was approved by the ethical committee of the Institution (ethic committee IDIAP Jordi Gol, file P14/134) and was conducted in accordance with the general principles for observational studies [15].

### Data sources

The Information system for the development of research in primary care of Catalonia (SIDIAP), [16] which compiles administrative data and clinical information contained in the Electronic PHCC’s medical records, was used to identify vaccinations, comorbidities and underlying risk conditions among study subjects and to establish baseline characteristics of cohort members at study start. We assumed that information in primary care clinical records was complete; so, a vaccination, comorbidity or risk condition was considered absent if it was not recorded. Quality criteria for clinical data of the SIDIAP research database has been reported elsewhere [17].

To identify study events (hospitalisations for pneumococcal and all-cause pneumonia) occurred among cohort members across study period, we used the national surveillance system for hospital discharge data (“Conjunto Minimo Basico de Datos”, CMBD) maintained by the Spanish Ministry of Health [18]. The CMBD system includes 98% of Spanish hospitals, covering an estimated 99.5% of the Spanish population (covered in the National Health Care System by a compulsory health insurance). In the present study we used CMBD hospital discharge codes, coded according to the International Classification of Diseases, 9th Revision, Clinical Modification (ICD-9), reported during 2015 from the 68 Catalonian hospitals.

### Data set linkage

The two data sets (SIDIAP and CMBD) were linked by *the Catalan Institute of Health (ICS)*, the public health provider, that acts as the trusted third part between *SIDIAP* and *CatSalut* (the Catalan Service of Health, whose have the hospital admissions).

The steps to made the linkage were as follows:

- *SIDIAP* provides the anonymous identifies of the cohort of the project to the ICS.
- The *ICS* translates the identify to the CIP, the Patient Identification Code, that is the unique identifier of a patient in Catalonia.
- *Catsalut* returns the hospital data related to those patients to the *ICS*.
- The *ICS* anonymizes back the IDs and get the data back to *SIDIAP*, this time including the hospital data.

The Catalan Institute of Health is the only that can made the linkage because they know the relation between the anonymous identifier and the open one (the CIP).

As the assignation of patients is done through the CIP, it is directly, is not a probabilistic assignation; so, there are not duplications. The only possible case is which one patient does not link with any CIP, and in this situation it will not have hospital admissions.

### Outcome definitions

Pneumococcal pneumonia (ICD-9: 481) and all-cause pneumonia (ICD-9: 480 to 487.0) were defined on the basis of hospital discharge codes (any diagnostic position) reported by the CMBD in hospitalisations occurred among cohort members from 01/01/2015 throughout 31/12/2015. Only a first episode of hospitalisation from pneumonia during the study period was considered; so, the analyses do not include multiple events per person. Death from any cause was considered according to administrative data (vital status), which is periodically updated in the SIDIAP data base. Death from pneumonia (case-fatality) was considered when the patient died (in-hospital or not) within the first 30-day after pneumonia diagnosis.

Because of the enormous size of the study cohort, an active following of study participants was considered not feasible. Nevertheless, all participating hospitals in the study setting basically apply similar diagnostic checklist and treatment for patients presenting with a clinical suspicion of pneumonia (which is established on the basis of an acute respiratory illness, with evidence of the presence of a new infiltrate in a chest radiograph). Conventional diagnostic workup included blood culture, sputum culture and *S. Pneumoniae* urinary antigen test (*Binax-NOW*), which were performed as indicated by the attending physician in each case.

### Exposure

The main explanatory variable was PCV13 vaccination status. It was determined by a review of the PHCCs’ electronic clinical records which contain specially designated fields for pneumococcal and influenza vaccinations (virtually all of them are administered at the PHCCs in the Spanish Health System). At the beginning of the study, cohort members were classified into the PCV13 vaccinated group if they had received at least one dose of PCV13 before the study started. Across the study period, PCV13 vaccination status was a time-varying condition given some individuals received the vaccine after the study start. Subjects were considered to be vaccinated 14 days after vaccine administration. For the PPsV23 vaccination status as covariate, cohort members were considered vaccinated if they had received the vaccine at any time. For influenza vaccination status, cohort members were considered as vaccinated if they had received the flu vaccine in the prior autumn.

### Covariates

Besides PPsV23 and influenza vaccine status, baseline covariates were age, sex, history of hospitalisation for pneumococcal disease or pneumonia during the previous 24-month, presence of chronic pulmonary/respiratory disease, chronic heart disease, diabetes mellitus, chronic liver disease, alcoholism, current smoking, and immunological situation. Immunocompromise was a composite variable defined by the presence of any one of the following: immunodeficiency/HIV infection, asplenia, chronic renal disease, bone marrow transplantation, cancer and/or immunosuppressive medication. Definitions used to identify comorbidities/underlying conditions are listed in the Appendix.

### Statistical analysis

Incidence rates were calculated as person-years, considering in the denominator the sum of the persons-time contributed to each individual during the study period. Baseline characteristics according to PCV13 vaccination status were compared using Chi-squared or T test as appropriate.

Cox regression models for time-varying covariables were used to evaluate the association between having received the PCV13 and the time of the first outcome during the study period [19]. Pneumococcal vaccination status was a time-varying condition, whereas the other covariates were defined at study entry. All abovementioned covariates were initially considered potential candidates for the calculation of multivariable Cox models. The method to select a subset of covariates to include in the final models was the purposeful selection [19]. The proportional hazard assumptions were assessed by adding the covariate by log-time interactions to the model. PPsV23 and influenza vaccine status were judged epidemiologically relevant covariates, being included in all the final models. All models were compared by the partial likelihood ratio test and Akaike information criterion.

Besides the main analysis including all study population, we performed four supplementary stratified analyses focused on middle-age individuals (50–64 years), elderly people (65 years or older), immunocompetent and immunocompromised persons.

All results were expressed with 95% confidence intervals (CIs). Statistical significance was set at *p* < 0.05 (two-tailed). The analyses were performed using Stata/SE 12.1 (Stata Corp.).

Rationale for sample size and statistical power: with an expected incidence of 7 cases per 1000 persons-year for all-cause pneumonia, considering a PCV13 coverage of 0.5%, with a *p* = 0.05 (two-tailed) the study cohort (*N* = 2,025,730 individuals followed during a one-year period) has a statistical power of 70% to detect a possible PCV13 effectiveness of 35% against this outcome.

## Results

The 2,025,730 cohort members were observed for a total of 1,990,701 person-years, of which 6912 person-years were PCV13 vaccinated. At study start, 5010 cohort members had received PCV13. Of the remaining 2,020,720 cohort members initially classified into the non-vaccinated group, 4460 received PCV13 later (contributing to the analyses with 2260 person-years in the non-vaccinated group and 2153 person-years in the vaccinated group).

At baseline, mean age of participants was 66 years-old (SD: 11.5) and 932,072 (46%) were male. PCV13 vaccinated subjects were older, suffered more comorbidities, and had a higher proportion of PPsV23 and influenza vaccination than PCV13 unvaccinated subjects (Table 1).

**Table 1:** Baseline characteristics of 2,025,730 cohort members according to their PCV13 vaccination status before the study started

	PCV13 VACCINATED N=5,010 n (%)	PCV13 UNVACCINATED N=2,020,720 n (%)	p value^a^
Age^b^			
50-64 years	1,393 (27.8)	1,015,783 (50.3)	<0.001
65-79 years	2,256 (45.0)	687,149 (34.0)	
≥ 80 years	1,361 (27.2)	317,788 (15.7)	
Sex, male	2,750 (54.9)	929,322 (46.0)	<0.001
History of pneumococcal disease in previous 2 yrs.	91 (1.8)	2,634 (0.1)	<0.001
History of all cause pneumonia in previous 2 yrs.	324 (6.5)	14,432 (0.7)	<0.001
PPV23 vaccination at any time	4,050 (80.8)	782,896 (38.7)	<0.001
Influenza vaccination in the prior autumn	4,143 (82.7)	679,345 (33.6)	<0.001
Chronic respiratory disease	1,605 (32.0)	209,916 (10.4)	<0.001
Chronic heart disease	1,553 (31.0)	245,929 (12.2)	<0.001
Diabetes mellitus	1,574 (31.4)	338,749 (16.8)	<0.001
Chronic liver disease	341 (6.8)	41,173 (2.0)	<0.001
Chronic renal disease	454 (9.1)	16,302 (0.8)	<0.001
Alcoholism	162 (3.2)	58,313 (2.9)	0.142
Smoking	465 (9.3)	315,613 (15.6)	<0.001
Immunocompromise^c^	2,109 (42.1)	173,945 (8.6)	<0.001
Multiple comorbidities			
Immunocompetent subjects	920 (18.4)	220,718 (10.9)	<0.001
Immunocompromised subjects	1,696 (33.9)	106,667 (5.3)	<0.001

^a^ p values were calculated with chi-square test.

^b^ At the beginning of the study, mean age of the unvaccinated and vaccinated subjects were 66.02 years (Standard Deviation, SD: 11.46) and 71.47 years (SD: 10.83) respectively.

^c^ Immunocompromise was a composite variable defined by the presence of any one of the following: cancer (solid organ or haematological neoplasia), chronic severe nephropathy (nephrotic syndrome, renal failure, dialysis or transplantation), anatomical or functional asplenia, immunodeficiency (including AIDS), and long-term corticosteroid therapy (20 mg/day of prednisone) or another immunosuppressive medication.

During the study period, 47,265 (2.3%) cohort members died and 14,488 (0.7%) moved or were lost patients. An amount of 12,699 cohort members had a first episode of hospitalisation for all-cause pneumonia, of which 1648 were pneumococcal pneumonia (Fig. 1).

Incidence rates (per 100,000 person-years) were 82.8 (95% CI: 77.7–88.1) for pneumococcal pneumonia (289.3 in PCV13 vaccinated vs 82.1 in PCV13 unvaccinated), 637.9 (95% CI: 599.0–678.7) for all-cause pneumonia (3298.6 in PCV13 vaccinated vs 626.6 in PCV13 unvaccinated) and 2367.2 (95% CI: 2222.8–2518.7) for all-cause death (5944.1 in PCV13 vaccinated vs 2354.5 in PCV13 unvaccinated).

Considering all-pneumonia cases, case-fatality was 8.3% (19/228) in PCV13 vaccinated vs 6.7% (836/12471) in PCV13 unvaccinated (*p* = 0.330). Considering pneumococcal pneumonia, case-fatality was 10% (2/20) in PCV13 vaccinated vs 4.9% (79/1628) in PCV13 unvaccinated (*p* = 0.290).

Table 2 shows absolute number of events, incidences and risks of hospitalisation for pneumococcal pneumonia, all-cause pneumonia and all-cause death according to PCV13 vaccination status in the total study population. In the unadjusted analyses, as well as in the age and sex-adjusted analyses, PCV13 was associated with an increased risk for all analysed outcomes. In the multivariable analyses, PCV13 vaccination did not alter significantly the risk of pneumococcal pneumonia (multivariable Hazard Ratio [mHR]: 1.17; 95% CI: 0.75–1.83; *p* = 0.493) or all-cause death (mHR: 1.07; 95% CI: 0.97–1.18; *p* = 0.190), but it remained significantly associated with an increased risk of all-cause pneumonia among vaccinated subjects (mHR: 1.69; 95% CI: 1.48–1.94; *p* < 0.001).

**Table 2:** **.** Incidence and Risk of hospitalisation for pneumococcal pneumonia, all-cause pneumonia and death from any cause in relation to PCV13 vaccination status in the total study population (N=2,025,730)

Event Parameter	Pneumococcal pneumonia	All cause pneumonia	Death from any cause
Number of events			
Vaccinated	20	228	420
Unvaccinated	1,628	12,471	46,845
Unadjusted incidence rate per 1000			
person-years			
Vaccinated	289.3	3,298.6	5,944.0
(95% CI)	(176.8-445.5)	(2,909.4-3,740.6)	(5,385.2-6,562.1)
Unvaccinated	82.1		
(95% CI)	(77.1-87.4)	628.6 (590.3-668.8)	2,354.5 (2,210.9-2,505.2)
Unadjusted hazard ratio	3.70	5.44	2.51
(95% CI)	(2.38-5.76)	(4.77-6.20)	(2.28-2.76)
p value	<0.001	<0.001	<0.001
Age and sex adjusted hazard ratio	2.40	3.42	1.68
(95% CI)	(1.55-3.74)	(3.00-3.89)	(1.53-1.85)
p value	<0.001	<0.001	<0.001
Multivariable-adjusted hazard ratio	1.17^a^	1.69^b^	1.07^c^
(95% CI)	(0.75-1.83)	(1.48-1.94)	(0.97-1.18)
p value	0.493	<0.001	0.190

NOTE: The hazard ratios are for PCV13 vaccinated subjects as compared with PCV13 unvaccinated subjects.

^a^adjusted for age (continuous), sex, history of pneumococcal disease or pneumonia during the previous 24 months, presence of chronic pulmonary/respiratory disease, chronic heart disease, diabetes, chronic liver disease, alcoholism, current smoking, immunodeficiency, HIV infection, chronic renal disease, cancer, immunosuppressive therapy, history of pneumococcal polysaccharide vaccination at any time and receipt or non receipt of influenza vaccine in prior autumn.

^b^ adjusted for age (continuous), sex, history of pneumococcal disease or pneumonia during the previous 24 months, presence of chronic pulmonary/respiratory disease, chronic heart disease, diabetes, chronic liver disease, alcoholism, current smoking, immunodeficiency, HIV infection, chronic renal disease, bone marrow transplantation, cancer, immunosuppressive therapy, history of pneumococcal polysaccharide vaccination at any time and receipt or non receipt of influenza vaccine in prior autumn.

^c^adjusted for age (continuous), sex, history of pneumococcal disease or pneumonia during the previous 24 months, presence of chronic pulmonary/respiratory disease, chronic heart disease, diabetes, chronic liver disease, alcoholism, current smoking, asplenia, immunodeficiency, HIV infection, chronic renal disease, bone marrow transplantation, cancer, immunosuppressive therapy, history of pneumococcal polysaccharide vaccination at any time and receipt or non receipt of influenza vaccine in prior autumn.

In stratified analyses according to age subgroups and immunological situation (Table 3), after multivariable adjustments the PCV13 vaccination did not shown any significant effect against pneumococcal pneumonia and all-cause death. However, it appeared significantly associated with an increased risk of all-cause pneumonia among elderly people (mHR: 1.76; 95% CI: 1.52–2.04; p < 0.001), immunocompromised persons (mHR: 1.51; 95% CI: 1.24–1.83; p < 0.001) and immunocompetent subjects (mHR: 1.86; 95% CI: 1.55–2.25; p < 0.001).

**Table 3:** Stratified analyses on PCV13 effectiveness according to age subgroups and immunological situation

	Age Strata		Immunological Status	
	50-64 years n= 1,017,176	≥65 years n=1,008,554	Compromised n=176,054	Competent n=1,849,676
Pneumococcal				
pneumonia				
Number of events	350	1,298	401	1,247
Multivariable HR	0.58	1.32	0.86	1.47
(95% CI)	(0.20-1.67)	(0.81-2.18)	(0.44-1.69)	(0.81-2.68)
p value	0.314	0.268	0.664	0.204
All cause pneumonia				
Number of events	2,115	10,584	3,222	9,477
Multivariable HR	1.21	1.76	1.51	1.86
(95% CI)	(0.85-1.72)	(1.52-2.04)	(1.24-1.83)	(1.55-2.25)
p value	0.283	<0.001	<0.001	<0.001
All cause death				
Number of events	4,984	42,281	14,615	32,650
Multivariable HR	1.01	1.06	1.01	1.10
(95% CI)	(0.77-1.32)	(0.95-1.17)	(0.88-1.14)	(0.95-1.27)
p value	0.951	0.309	0.928	0.217

The HRs (hazard ratios) are for PCV13 vaccinated subjects as compared with PCV13 nonvaccinated and were adjusted, where appropriate, for age, sex, history of pneumococcal disease or pneumonia during the previous 24 months, presence of chronic pulmonary/respiratory disease, chronic heart disease, diabetes, chronic liver disease, alcoholism, current smoking, asplenia, immunodeficiency, HIV infection, chronic renal disease, bone marrow transplantation, cancer, immunosuppressive therapy, history of pneumococcal polysaccharide vaccination at any time and receipt or non receipt of influenza vaccine in prior autumn

## Discussion

Clinical benefits using PCV13 in adults are controversial [8–11]. We undertook a large population-based cohort study to investigate the clinical effectiveness of PCV13 vaccination in preventing hospitalisations for pneumococcal and all-cause pneumonia among middle-aged and older adults in Catalonia. To our knowledge, apart from the *CAPITA* trial, [8] this is the first large prospective study evaluating this issue in practice.

As main findings, PCV13 vaccination did not emerge effective to prevent pneumonia and/or death in our study population. In the initial unadjusted analyses, crude incidence rates for all analysed outcomes were largely greater in PCV13 vaccinated than in PCV13 unvaccinated subjects, reflecting the baseline excess risk of vaccinated (who had higher prevalence of underlying risk conditions than unvaccinated subjects). After multivariable adjustments we found that PCV13 vaccination did not alter significantly the risk of hospitalisation for pneumococcal pneumonia and/or all-cause death, but it remained significantly associated with an increased risk of hospitalisation for all-cause pneumonia (mHR: 1.69; 95% CI: 1.48–1.94) considering the total study cohort. In stratified analyses focused on immunocompromised persons and elderly individuals, main target groups where PCV13 is currently recommended, [4, 5] the PCV13 did not emerge effective either. In fact, excluding middle-aged individuals, PCV13 vaccination appeared significantly associated with an increased risk of all-cause pneumonia in all analysed strata.

Although apparently opposite, our data does not fully disagree with data reported in the *CAPITA* trial (the unique RCT that has evaluated the clinical efficacy of the PCV13 in adults to date) [8]. Concretely, the *CAPITA* study (large RCT comparing PCV13 vs placebo among 84,496 individuals ≥65 years in the Netherlands) reported a significant PCV13 efficacy of 45.6% (95% CI: 21.8% to 62.5%) against vaccine-type pneumococcal pneumonia but did not show any significant efficacy of PCV13 against all-cause pneumonia, death from pneumonia and all-cause death [8]. The reported PCV13 efficacy against all-type pneumococcal pneumonia was 30.6% (95% CI: 9.8% to 46.7%) in the CAPITA trial, [8] which overlaps with data observed in the present study (mHR: 1.17; 95% CI: 0.75–1.83). On other hand, considering all-cause pneumonia, we note that a null or negative effect of PCV13 against all-cause community-acquired pneumonia was not fully excluded among the general elderly population in the CAPITA study (PCV13 efficacy: − 5%to 14%) [8].

We highlight that comparison is difficult due to important methodological and epidemiological differences between both studies. Indeed, while no routine antipneumococcal vaccination for adults and/or children had been introduced in the Netherlands when the CAPITA trial started, [20] the PPsV23 for adults and the PCV7/PCV13 for children had been used in Catalonia since the 2000s [12, 13]. Thus, the PCV13 efficacy estimates in a population with an expected greater prevalence of circulating PCV13 serotypes can not be fully compared with the PCV13 effectiveness in a population with a minor prevalence of circulating PCV13-serotypes (if a herd effect from PCV’s childhood immunisation may be expected) [21].

At present, because the risk of immunization is believed to be very small, many experts and clinicians recommend pneumococcal vaccination for all persons with an increased risk of infection or death (i.e, high-risk adults and elderly persons) [4, 5, 22, 23]. However, we note that having an increased risk of suffering a pneumococcal infection (e.g. immunocompromising or other risk conditions) does not necessarily imply that vaccination of these persons will be an effective measure in the practice [24–26]. According to our results, the possibility of a harm effect of PCV13 vaccination (increasing the risk of all-cause pneumonia) may not be completely excluded. Hypothetically, since PCV13 vaccination may reduce pneumococcal carriage/colonization and this could alter the nasopharyngeal/respiratory tract flora, an increase in infections by other microorganisms (especially in high-risk or elderly individuals) could be a possible explanation for this unexpected finding.

The authors underline the importance of RCT data, and note the limited amount of such data regarding clinical efficacy of PCV13 in adults. Clinical outcomes of patients are affected by many factors, being antibody production and associated phagocyte stimulation only one aspect, which incompletely represent the immune response overall (especially aging immune response) and may not adequately reflect the potential impact of the vaccine on clinical outcomes [2, 3, 27].

Major strengths in this study were the large size and representativity of the study cohort (involving more than 2 million people, almost 80% of overall population over 50 years in Catalonia), and the use of survival analysis methods to estimate PCV13 effectiveness against public healt relevant outcomes such as hospitalisation from pneumococcal or all-cause pneumonia and all-cause death. Although it is not a RCT, the large size of the study population together with the adjustment of major possible confounding variables (e.g, history of previous pneumococcal disease or pneumonia, comorbidities and underlying risk conditions, PPsV23 and influenza vaccination status) in the multivariable analyses may provide an acceptable basis to assess the potential effects of PCV13 vaccination in adults at present.

Major limitations in this study are related with its observational nature: mainly, the non-randomised vaccination and the scarce PCV13 coverage in the study population (which limits statistical power to evaluate vaccination effectiveness against uncommon events, especially in subgroups analyses). The authors recognize these inherent limitations but note that, opposite to “vaccine efficacy” that must be evaluated by trials with controlled conditions, “vaccination effectiveness” may be evaluated by observational studies conducted in the real-practice conditions (i.e., study populations where the vaccine is not routinely used or it is more frequently prescribed for persons with underlying risk conditions as in the present study).

In this study, the large differences in outcomes between PCV13 vaccinated and unvaccinated suggest that vaccinated persons were sicker. The large differences observed in crude incidence rates between vaccinated and unvaccinated mostly resolved in the multivariable analysis, although a residual confounding due to unobserved factors (as all observational studies) may not be completely excluded [19]. In favour of a non important residual confounding in the final PCV13 effectiveness estimates, we note that the adjusted risk of all-cause death did not significantly differ between vaccinated and unvaccinated (mHR: 1.07; 95% CI: 0.97–1.18; *p* = 0.190), despite crude mortality had been more than double in vaccinated (5944 per 100,000) than in unvaccinated (2354 per 100,000).

Our study is not able to assess the possible overall effect of PCV13 against all pneumonia (including outpatient cases) because only hospitalisations from pneumonia were included. We note that pneumonia cases managed outside the hospital could represent approximately a 25% of overall pneumonia cases in older adults and this was not evaluated in the present study.

In the present study, comorbidities/underlying risk conditions for pneumococcal disease were considered only at baseline. Incident comorbidities occurred after study start were not assessed, which could affect vaccination effectiveness estimates for the subgroup of patients who developed later any of these comorbidities/risk conditions considered as an indication for PCV13 vaccination. Basically, this could affect to immunocompromising conditions (although their expected incidence would be small). We note that the majority of comorbidities/risk conditions related to PCV13 indication are prevalent conditions (mainly chronic illnesses), which were established at baseline and little incidence of new cases would be expected throughout one-year period after study start.

On other hand, although the validity of clinical data source was previously checked, [16] information bias may have occurred if some vaccinations and/or comorbidities/underlying conditions were not recorded. We do not have available data for pneumococcal serotypes (which is not reflected in the Spanish CMBD system) and, consequently, vaccination effectiveness against vaccine-type pneumococcal infections (the most specific outcome evaluating vaccine efficacy) can not be assessed in the present study. We note, however, that the expected incidence of this outcome is low (10.2 cases per 100,000 elderly population-year according to laboratory-based epidemiological data in Catalonia during 2014) [28] and underline the fact that our study provides other very important data from a clinical and public health point of view (such as hospitalisation for pneumococcal and all-cause pneumonia, death from pneumonia and death from any cause).

## Conclusions

In the present population-based cohort study involving more than 2 million people over 50 years old in Catalonia, clinical benefits from PCV13 vaccination have not emerged. Apart from a possible protective effect of the PCV13 against vaccine-type infections (which can not be assessed in the present study), our unadjusted and adjusted data show that PCV13 vaccination did not provide clinical benefits in reducing hospitalisation from overall pneumococcal pneumonia, as in the general people over 50 years old as well as in immunocompromised subjects and elderly individuals (main targeted groups where PCV13 is currently recommended in adults). Our data does not exclude a possible null or negative effect (increasing risk of all-cause pneumonia) among PCV13 vaccinated subjects, which should be closely surveilled in future years.

We note that this study has been made in a single geographical region, Catalonia, with intermediate/high PCV13 and PPsV23 uptakes in children and older adults, respectively, [12, 13] and moderate incidence of pneumococcal infections [28]. Logically, PCV13 impact/effectiveness may vary in other geographical settings depending on multiple factors (such as susceptibility for pneumococcal infections among the population, prevalence of distinct circulating serotypes, PPsV23 coverage, routine use of PCV13 in children, possible herd effects, etc).

We emphasize that our findings must be interpreted with caution because PCV13 coverage was low, vaccination was not randomised and time follow-up was limited. Further investigations involving more vaccinated person-years at-observation are needed to confirm or reject these early results.

## Appendix

### Criteria used to identify comorbidities and underlying risk conditions in the study population.

The following comorbidities and underlying risk conditions were established according to the presence of ICD-10 codes [International Classification of Diseases, 10th Revision] registered in the electronic primary medical records of each cohort member at baseline:

- **Chronic pulmonary/respiratory disease**: it included chronic bronchitis/emphysema (J41-J44), asthma (J45-J46) and/or other chronic pulmonary diseases (P27, E84, J47)
- **Chronic heart disease**: it included congestive heart failure (I50), coronary artery disease (I20-I22, I25) and/or other chronic heart diseases (I05-I08, I11,I35-I37,I42, I51.7)
- **Diabetes mellitus** (E10-E14).
- **Chronic liver disease**: it included chronic viral hepatitis (B18), cirrhosis (K74) and/or alcoholic hepatitis (K70))
- **Alcoholism** (F10, G31.2, G62.1, G72.1, I42.6, K29.2, K70)
- **Smoking** (F17).
- **Anatomic or functional asplenia** (D57, D73, Q89)
- **Primary immunodeficiency** (D80-D84)
- **HIV infection** (B20-B24)
- **Chronic renal disease**: it included nephrotic syndrome (N04, N39.1) and severe chronic renal failure (N18-N19 with glomerular filtration rate ≤ 30 ml/min)
- **Bone marrow transplantation** (Z94)
- **Cancer**: it included solid organ or haematological neoplasia (C00 to C97) diagnosed within previous 5 years.
- **Immunosupresive therapy**: it included long-term immunosuppressive medication and/or radiotherapy in the previous 12 months (coded according to specific SIDIAP codes).

## Abbreviations

ACIP: Advisory Committee on Immunization Practices
CAPITA: Community-Acquired Pneumonia Immunization Trial in Adults
CDC: Centers for Disease Control and Prevention
CI: Confidence interval
CMBD: Conjunto Minimo Basico de Datos
EPIVAC: Effectiveness of Pneumococcal and Influenza Vaccinations among Adults in Catalonia
ICD: International Classification of Diseases
ICS: Institut Catala de la Salut
mHR: multivariable Hazard Ratio
PCV13: 13-valent protein-polysaccharide conjugate vaccine
PHCCs: Primary Health Care Centres
PPsV23: 23-valent pneumococcal polysaccharide vaccine
RCT: randomized controlled trial
SD: Standard deviation
SIDIAP: Information system for the development of research in primary care of Catalonia
